# Apoptotic circulating tumor cells (CTCs) in the peripheral blood of metastatic colorectal cancer patients are associated with liver metastasis but not CTCs

**DOI:** 10.18632/oncotarget.1524

**Published:** 2013-10-17

**Authors:** Joshua E. Allen, Bikramajit Singh Saroya, Miriam Kunkel, David T. Dicker, Avisnata Das, Kristi L. Peters, Jamal Joudeh, Junjia Zhu, Wafik S. El-Deiry

**Affiliations:** ^1^ Penn State Hershey Cancer Institute, Penn State College of Medicine, Hershey, PA

**Keywords:** CTC, colon cancer, colorectal cancer, metastasis, prognostic marker, apoptosis

## Abstract

Enumeration of circulating tumor cells (CTCs) by the CellSearch system provides prognostic information in metastatic colorectal cancer, regardless of metastatic site. We found that CTCs generally represent <1% of observed events with CellSearch analysis and adapted scoring criteria to classify other peripheral blood events. Examination of twenty two metastatic colorectal cancer patients' blood revealed that patients with high CEA or liver metastases, but not lung or distant lymph node metastases, possessed significant numbers of apoptotic CTCs prior to treatment initiation by Fischer's exact test. Six out of eleven patients with liver metastasis possessed apoptotic CTCs whereas one of nine patients with other metastases had measurable apoptotic CTCs. An elevated CTC number was not necessarily associated with apoptotic CTCs or CTC debris by Spearman's correlation, suggesting the metastatic site rather than CTCs per se as contributing to the origin of these events.

## INTRODUCTION

The detection of metastatic cells in the blood (circulating tumor cells (CTCs)) as a non-invasive window to monitor disease progression and prognosis as well as provide disease-specific information to aid in therapeutic stratification is paramount. Colorectal cancer has a 5-year survival rate of ~65% with metastasis being the major determinant of outcome [1]. Thus the early and dynamic detection of metastasis in colorectal cancer has gained significant impetus following the relatively recent emergence of detection technologies. The CellSearch system is a semi-automated method to identify tumor cells of epithelial origin in the blood and is FDA-approved as a prognostic marker for metastatic colorectal, breast, and prostate cancers. Metastatic colorectal cancer patients with >3 CTCs in 7.5 mL of peripheral blood have a significantly worse progression-free survival and overall survival than patients below this threshold [2, 3]. A number of technologies have emerged to compete with the CellSearch system following its approval by the FDA, including techniques that detect circulating nucleic acids or filter tumor cells based on size or electrostatic properties rather than relying on epithelial cell adhesion molecule (EpCAM) expression [4]. The latter is especially important as it allows for the capture of live cells. There is also an increasing appreciation for the involvement of epithelial-mesenchymal transition (EMT) in CTCs [5, 6], though the requisite loss of EpCAM expression in EMT and CTCs remains unclear.

Scoring an identified peripheral blood event as a CTC using the CellSearch criteria requires the event to be a single cell, possess an appropriate morphology, express EpCAM and cytokeratin, and lack CD45 that is expressed on the surface of leukocytes. In spite of this single cell definition, clusters of CTCs, also called tumor cell microemboli, have been reported using the CellSearch system as well as other devices [7, 8]. A study in small cell lung cancer found that tumor cell microemboli as well as apoptotic CTCs were independent prognostic factors, demonstrating that cellular bodies other than CTCs are indeed of clinical significance [9]. While conducting clinical and research assays using the CellSearch system we previously observed a number of events that did not meet the criteria for scoring as a CTC and were clearly not leukocytes, but rather appeared to represent apoptotic cells, dead cells, or cellular debris. We hypothesized that treatment initiation in metastatic colorectal cancer patients would result in a decrease in CTCs and an increase in apoptotic CTCs and cellular debris. We adapted a previously reported scoring system for these unreported peripheral blood events identified by the CellSearch system [10]. Following refinement of the criteria to optimize reviewer concordance, we investigated the prevalence and correlational significance of these events in metastatic colorectal cancer patients before and after treatment initiation.

## RESULTS

### Patient Characteristics

33 patients were enrolled between March 2011 and November 2012, 22 of which were included in this study as eligible for evaluation of the study objectives. Patient characteristics, including disease histology and genetics, are provided in Table 1.

**Table 1:** Patient and disease characteristics Patients were excluded from analysis if they were withdrawn during the study duration for any reason, lack a baseline sample, or lack a sample following cycle 1 initiation.

Variable	Descriptive Statistics (Total n=22)
Gender Male Female	14 (64%) 8 (36%)
Age Mean(SD) Median (Range)	63.5 (15.3) 68.5 (27 – 88)
Primary Location Rectum Colon	(n= 2 missing) 8 (40%) 12 (60%)
Primary Histology Adenocarcinoma With signet ring cell features With mucinous features	(n= 2 missing) 16 (80%) 1 (5%) 3 (15%)
Primary Differentiation Well or moderate Poor or differentiated	(n= 4 missing) 14 (78%) 4 (22%)
Liver Metastasis Yes No	(n= 2 missing) 11 (55%) 9 (45%)
Lung Metastasis Yes No	(n= 2 missing) 10 (50%) 10 (50%)
T 2 3 4	(n= 10 missing) 1 (8%) 5 (42%) 6 (50%)
N 0 1 2	(n= 10 missing) 1 (8%) 1 (8%) 10 (83%)

Variable	Descriptive Statistics (Total n=22)
Lymphatic Invasion Yes No	(n= 11 missing) 7 (64%) 4 (36%)
Venous Invasion Yes No	(n= 12 missing) 5 (50%) 5 (50%)
Perineural Invasion Yes No	(n= 11 missing) 4 (36%) 7 (64%)
KRAS WT G12A G12V	(n= 5 missing) 15 (88%) 1 (6%) 1 (6%)
BRAF WT V600E	(n= 6 missing) 14 (88%) 2 (12%)
EGFR Expression Mean(SD) Median (Range)	(n= 10 missing) 2.9 (1.8) 2.1 (1.1 – 6.5)
ERCC1 Expression Mean(SD) Median (Range)	(n= 10 missing) 1.2 (0.7) 1.1 (0.4 – 3.1)
TS Expression Mean(SD) Median (Range)	(n= 10 missing) 3.6 (1.9) 3.6 (1.5 – 7.0)
CEA Mean(SD) Median (Range)	(n= 2 missing) 294.6 (1104.4) 7.6 (1.6 – 4967.7)
Tumor-Liver volume ratio Mean(SD) Median (Range)	(n=13 missing) 0.2 (0.3) 0 (0 – 0.7)

### Categorizing peripheral blood events identified by the CellSearch system

**Figure 1:**
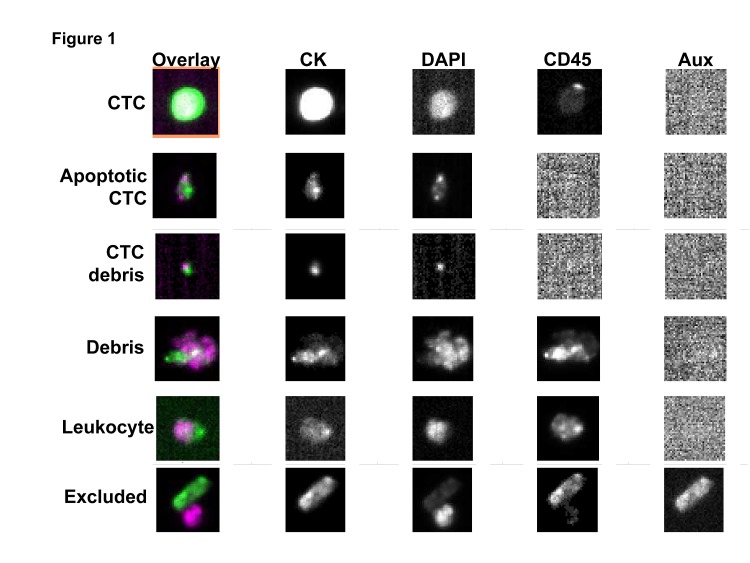
Exemplary images of peripheral blood event categories

To evaluate peripheral blood events other than CTCs, we first established a criteria for scoring general categories of events detected on the CellSearch system as one of the following: CTC, apoptotic CTC, CTC debris, debris, or leukocyte. Scoring of a training data set by 3 independent reviewers revealed a modest concordance that prompted revision of the category definitions (Figure S1A). The scoring criteria was refined and implemented to score a different training data set. This revised scoring criteria yielded an acceptable concordance among the independent reviewers and was used for analyzing the test data set (Figure 1; Figure S1B; Table 2).

**Table 2:** Categorization of peripheral blood events Events are excluded if there is evidence of identical staining images in all channels, staining in the auxiliary channel, or if 75% of the CK-PE and/or DAPI staining is not within the event image frame. For multiple bodies within a single event image, the bodies are scored as one event if the CK staining is touching and one of the bodies is <4 um. If both bodies are >4 um and the CK staining is touching, the bodies are scored as separate events.

Category	Description
CTC	DAPI positive, CK-PE positive, CD45-APC negative with round to oval nucleus, CK-PE staining is continuous, round, oval, or elongated, and 50% of the DAPI staining must be within the CK-PE staining.
Apoptotic	DAPI positive, CK-PE positive, CD45-APC negative, CK-PE that is exclusively punctuate
	Punctate or fragmented DAPI, CK-PE positive, CD45 APC negative
	DAPI positive, CK-PE positive, CD45-APC negative with round to oval nucleus, CK-PE staining that is <50% of the DAPI staining must be within the CK-PE staining.
CTC debris	CK-PE positive, CK-PE staining less than 4 μm in diameter, CD45-APC negative, and DAPI is less than 50% within the CK but still touching
Leukocytes	DAPI positive, CD45-APC positive events with round to oval morphology and a nucleus inside the CD45-APC staining.
Debris	Anything other than cells of interest, usually aggregates of staining reagents or debris in the sample or destroyed cells. Limit of 1 debris event per frame.

### Peripheral blood events before and after treatment

**Figure 2:**
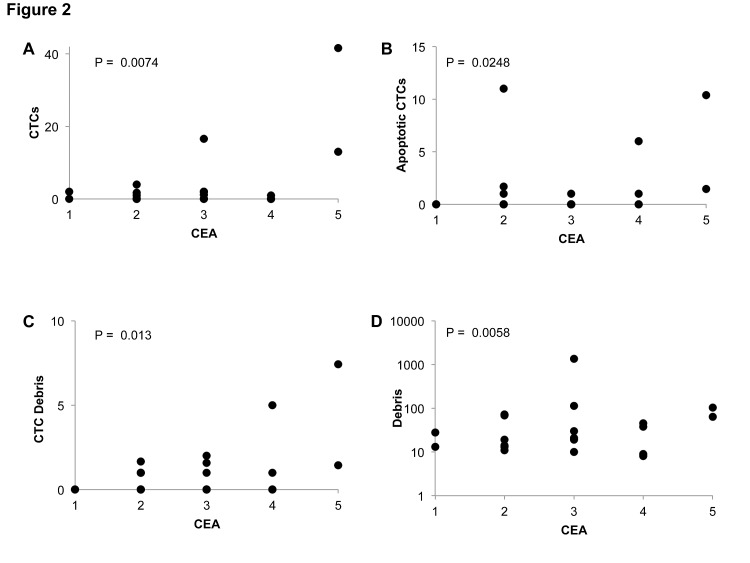
Correlation of peripheral blood events with CEA metastatic colorectal cancer patients before treatment initiation Peripheral blood event correlations before cycle 1 of treatment. CEA is categorized as follows: 1: <2.5 ng/mL, 2: 2.5-5 ng/mL, 3: 5-50 ng/mL, 4: 50-200 ng/mL, 5: >200 ng/mL.

Using the peripheral blood events scoring system we evaluated CellSearch data from the twenty two patients at prior to cycle 1, 1-week following cycle 1 initiation, and prior to cycle 2 of therapy (Figure 2; Table 3). At baseline, four patients had >3 CTCs, three patients had >3 apoptotic CTCs, and two patients had >3 CTC debris. Debris was ubiquitously noted among patients at baseline, with the median count being 25 events. Following treatment initiation, CTCs dramatically decreased in nearly all patients at either follow-up time points. Surprisingly, we observed approximately the same or lower prevalence of apoptotic CTCs, CTC debris, and debris in the post-treatment samples as observed at baseline.

**Table 3:** Peripheral blood events in metastatic colorectal cancer patients before and after treatment Time point 1 is prior to cycle 1, time point 2 is 1 week following initiation of cycle 1, and time point is prior to cycle 2.

		Time Point 1	Time Point 2	Time Point 3
CTC	Mean(SD) Median (range)	4 (9.5) 0.5 (0-42)	1 (1.5) 0 (0-6)	1.1 (2.6) 0 (0-11)
Apoptotic CTC	Mean(SD) Median (range)	1.5 (3.2) 0 (0-11)	1.8 (2.3) 1 (0-8)	1.3 (2) 0 (0-5)
CTC debris	Mean(SD) Median (range)	1.1 (1.8) 0.5 (0-7)	1 (1.8) 0 (0-8)	0.7 (1.5) 0 (0-6)
Leukocytes	Mean(SD) Median (range)	30.5 (71.7) 13 (3-349)	19 (20.5) 14.5 (0-97)	32 (31.4) 16 (2-103)
Debris	Mean(SD) Median (range)	96.1 (284.6) 25 (8-1363)	45.8 (60.9) 24 (5-253)	29.4 (30.5) 20 (1-117)
Excluded	Mean(SD) Median (range)	36.2 (32.8) 24.5 (7-150)	38.6 (34.4) 26.5 (10-151)	26.7 (18.2) 23 (6-74)
Total	Mean(SD) Median (range)	169.5 (384.4) 70 (34-1878)	107.1 (91.9) 83.5 (26-387)	91.2 (65.9) 69 (21-271)

### Relationships between peripheral blood events and baseline CEA or metastatic sites

**Figure 3:**
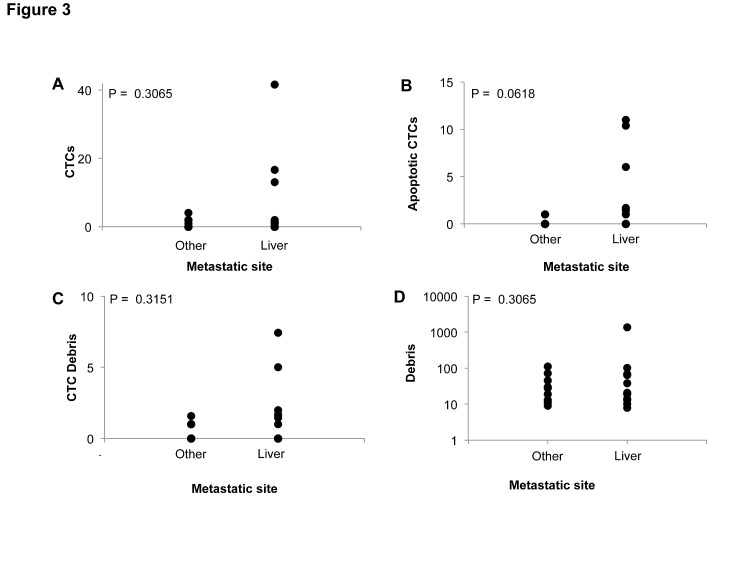
Correlation of peripheral blood events with sites of metastasis in metastatic colorectal cancer patients before treatment initiation Peripheral blood event correlations before cycle 1 of treatment.

We examined the relationships between peripheral blood events and carcinoembryonic antigen (CEA), which is one of the most commonly implemented and established prognostic markers in colorectal cancer. For statistical analysis, we created a binary categorical cutoff value for each blood event category, i.e. high or low group classification. Cutoff values were selected as the mean across the study population for each category except for CTCs, which was selected as 3 based on the FDA approved method. We found that patients with higher CEA, which is associated with a worse prognosis, generally possessed higher amounts of CTCs, apoptotic CTCs, and CTC debris (Figure 2). Patients with liver metastases also generally possessed higher levels of CTCs, apoptotic CTCs, and CTC debris, though apoptotic CTCs were the only association that approached statistical significance by Fischer's exact test (Figure 3). Furthermore, these associations with CEA as well as liver metastasis were only observed at baseline and not at 1-week post treatment in cycle 1 or prior to cycle 2 of therapy (Figure S2).

Given the common associations observed with CEA and liver metastasis, we analyzed Spearman's correlations to assess if peripheral blood events were associated with each other. Surprisingly, no association was found between these categorical events at prior to cycle 1 or cycle 2 of therapy (Table S1). However, CTCs and debris were found to have a significant positive correlation at 1-week post-treatment in cycle 1. 8 out of 11 patients with liver metastasis possessed apoptotic CTCs at 1 week following treatment initiation. Among these 8 patients that possessed apoptotic CTCs, 4 of these patients had no detectable CTCs prior to or 1 week following treatment.

## DISCUSSION

Successfully isolating and enumerating tumor cells in the blood represents a momentous achievement given the scarcity of this population. We and other investigators have also utilized this detection technology to investigate other biological fluids such as cerebrospinal fluid that are of prognostic significance [11, 12]. There is a movement to advance beyond counting CTCs that has arisen in parallel with the increasing appreciation of pronounced tumor heterogeneity, culminating in efforts to interrogate the molecular profile of CTCs in an effort to better understand and treat metastatic disease. Indeed markers of cancer stem cells [13, 14], EMT [5, 6], and other cell lineage markers have been explored in CTCs along with predictive markers of therapeutic response such as Her2 [15, 16].

While this report is limited to enumeration, our report investigates tumor-associated events in the blood beyond strictly defined CTCs. We initially hypothesized that treatment would decrease the amount of CTCs, which we predicted would undergo cell death to give rise to an increased prevalence of apoptotic CTCs, CTCs debris, and debris. While we observed a decrease in CTCs in response to treatment as predicted, we did not observe an increase in the other event categories. This observation suggests that CTCs may not give rise to these other peripheral blood events as measurable events one week after chemotherapy exposure and instead posits that perhaps apoptotic CTCs, CTC debris, and debris at baseline in the absence of chemotherapy exposure originate from the metastatic or primary site rather than CTCs. This is in full accordance with a prior study in small cell lung cancer that found that apoptotic CTCs were associated with more advanced disease and a worse overall survival and progression-free survival [9].

We observed that patients with liver metastases specifically possessed higher levels of CTCs, apoptotic CTCs, CTC debris, and debris. This suggests that these tumor-associated events are related to the metastatic site rather than the primary tumor. This notion is supported by the general lack of correlation amongst these tumor-associated events. Furthermore, the increased prevalence of apoptotic CTCs, CTC debris, and debris in patients with liver metastases agrees with the prior observation that small cell lung cancer patients with liver metastases have a much higher prevalence of apoptotic CTCs compared to patients with other sites of metastasis [9].

While our findings were statistically significant, this pilot study examined samples from 22 patients, which is a limited sample size. Investigation of these tumor-associated events in a larger cohort of metastatic patients will be needed to corroborate our observations that apoptotic CTCs, CTC debris, and debris are associated with liver metastasis and not CTCs. The heterogeneity of the patient population, including divergent prior treatments and disease genetics, must also be considered. Lastly, a sufficient but modest reviewer concordance was achieved with our revised criteria. An automated system using software algorithms has been recently reported for evaluating CTCs and other events identified by the CellSearch that may increase throughput and decrease bias [10].

Our findings extend the utility of the CellSearch system beyond enumerating CTCs to include other tumor-associated events. These observations indicate that apoptotic CTCs, CTC debris, and debris are also useful liquid biopsy markers that can be used to interrogate metastatic disease, particularly with respect to the liver, but may not be an effective pharmacodynamic marker like CTCs. Furthermore, these clinical data provide unanticipated insight into the origin of these tumor-associated events. This report highlights a potential advantage of cell-based assays over nucleic acid-based techniques that have been recently found to have a greater dynamic range and responsiveness [17].

## METHODS

### Study Design

The trial consented and enrolled 33 metastatic colorectal cancer patients at Penn State Hershey Medical Center to evaluate prognostic markers in CTCs and was approved by the Institutional Review Board at Penn State Hershey Medical Center (ClinicalTrials.gov Identifier: NCT01286883). Primary eligibility requirements were patients with metastatic colorectal cancer, >18 years of age, ECOG performance status of 0-3, no therapy 5 weeks prior to enrollment, no concurrent active solid malignancies, and a life expectancy of >6 weeks. Patient data were collected and managed using REDCap electronic capture tools hosted at Penn State University. Blood samples were collected at several intervals during the patient's chemotherapeutic regimen (FOLFOX/FOLFIRI or XELODA), including prior to cycle 1, 1 week following initiation of cycle, and prior to cycle 2. For blood sample collection, at least 7.5 mL of peripheral blood was collected into a CellSave tube and processed using the standard operating procedures for the CellSearch system (Veridex, LLC, Raritan, NJ).

### Enumeration of peripheral blood events

The CellSearch Analyzer II software pre-selects events that are potential CTCs based on EpCAM and CK positivity in close proximity to a DAPI signal. Pre-selection of these candidate CTC events do not take in to account CD45 staining or morphology and therefore, many of these events do not meet the definition of CTCs. The standard CellSearch procedure for scoring an event as a CTC requires interpretation by a trained operator. Candidate CTCs were scored by three reviewers. Our criteria for the peripheral blood event categories were designed by two reviewers based on a previous report [10], prior experience, and reviewing research samples as a training data set not scored in the validation or test data sets. Following an initial review of a training data set (Figure S1A), the criteria were refined as reported in Table 1. These refined criteria were used for all subsequent analyses, including the second validation data set and the test data set (Figure S1B). The concordance data sets were generated with three independent reviewers. Two independent blinded reviewers scored the test data set. For samples containing >150 events, 100 events were evaluated and extrapolated based on the total number of events. These 100 events were evaluated by 10 consecutive events spread evenly across the entire data set to ensure widespread sampling across the cartridge.

### Statistical analysis

Basic descriptive summary statistics (such as mean, median, standard deviation for continuous/count measures, and proportion for categorical measures) were generated to describe the peripheral blood events and patients' characteristics. Spearman correlation coefficients were used to examine the association between peripheral blood events for a given time point. Associations between peripheral blood events and patients' characteristics were studied by nonparametric Kruskal Wallis test or Fisher's Exact test when appropriate. For statistical analysis of peripheral blood events, the following cutoffs were used to generate high/low groups: CTC – 3, apoptotic CTCs – 2, CTC debris – 1, debris – 56. The CTC cutoff was selected based on the FDA approved method whereas the other cutoffs were the mean for that event across the study population. All analyses were performed using statistical software SAS version 9.3 (SAS Institute, Cary, NC).

## SUPPLEMENTARY FIGURES AND TABLES


