# Supplementary material for: Apoptotic circulating tumor cells (CTCs) in the peripheral blood of metastatic colorectal cancer patients are associated with liver metastasis but not CTCs
Source: Oncotarget. 2013 Oct 17;5(7):1753–60. doi: 10.18632/oncotarget.1524 (PMC4039127; doi:10.18632/oncotarget.1524)
Supplement: Supplementary file 1 [file oncotarget-05-1753-s001.docx]

**Supplementary Information**

**Apoptotic CTCs in the peripheral blood of metastatic colorectal cancer patients are associated with liver metastasis but not CTCs**

Joshua E. Allen, Bikram Singh Saroya, Miriam Kunkel, David T. Dicker, Avisnata Das, Kristi L. Peters, Jamal Joudeh, Junjia Zhu, and Wafik S. El-Deiry

Penn State Hershey Cancer Institute, Penn State College of Medicine, Hershey, PA USA

**Contents**

Figure S1. Concordance of independent blinded reviewers in training data sets.

Figure S2. Correlation of peripheral blood events with CEA and metastatic sites in metastatic colorectal cancer patients during treatment.

Table S1. Spearman correlation coefficients for peripheral blood events before and after treatment in metastatic colorectal cancer patients.

**Figure S1. Concordance of independent blinded reviewers in training data sets.** (A) Scoring of a training data set by 3 independent reviewers using initial scoring criteria. (B) Scoring of a different training data set by 3 independent reviewers with refined scoring criteria shown in Table 1.

**
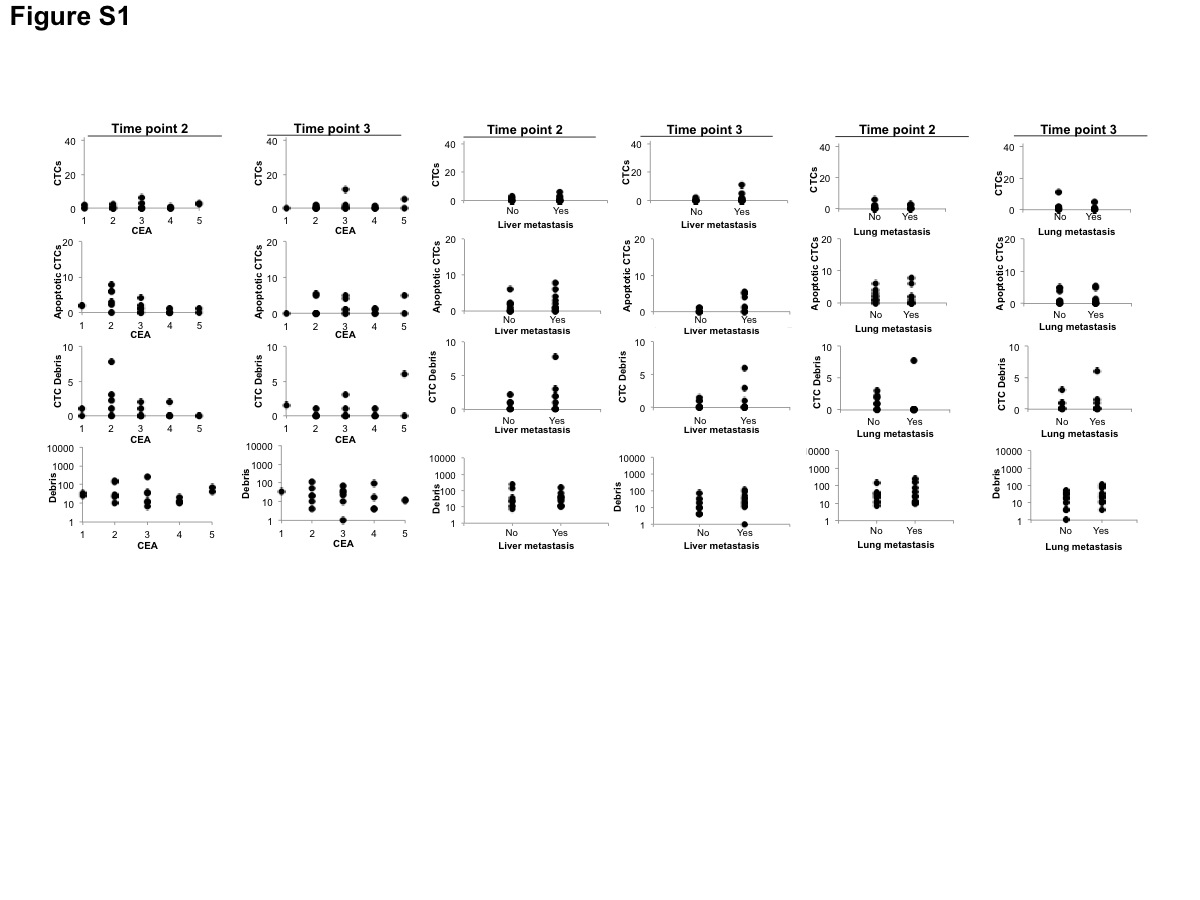
**

**Figure S2.** **Correlation of peripheral blood events with CEA and metastatic sites in metastatic colorectal cancer patients during treatment.** CEA is categorized as follows: 1: <2.5 ng/mL, 2: 2.5-5 ng/mL, 3: 5-50 ng/mL, 4: 50-200 ng/mL, 5: >200 ng/mL.

**Table S1. Spearman correlation coefficients for peripheral blood events before and after treatment in metastatic colorectal cancer patients.**

| **Time Point** | **Category** |  | **CTC** | **Apoptotic CTC** | **CTC debris** | **Debris** |
| --- | --- | --- | --- | --- | --- | --- |
| **C1D1** | **CTC** | Coefficient | 1 | 0.16027 | 0.33748 | 0.19649 |
|  |  | P value |  | 0.4762 | 0.1245 | 0.3808 |
|  | **Apoptotic CTC** | Coefficient | 0.16027 | 1 | 0.55217 | 0.06384 |
|  |  | P value | 0.4762 |  | 0.0077 | 0.7778 |
|  | **CTC debris** | Coefficient | 0.33748 | 0.55217 | 1 | -0.2034 |
|  |  | P value | 0.1245 | 0.0077 |  | 0.364 |
|  | **Debris** | Coefficient | 0.19649 | 0.06384 | -0.0445 | 1 |
|  |  | P value | 0.3808 | 0.7778 | 0.8441 |  |
| **C1D7** | **CTC** | Coefficient | 1 | -0.1368 | 0.20853 | 0.53284 |
|  |  | P value |  | 0.5438 | 0.3517 | 0.0107 |
|  | **Apoptotic CTC** | Coefficient | -0.1368 | 1 | 0.28138 | 0.28329 |
|  |  | P value | 0.5438 |  | 0.2046 | 0.2014 |
|  | **CTC debris** | Coefficient | 0.20853 | 0.28138 | 1 | 0.12245 |
|  |  | P value | 0.3517 | 0.2046 |  | 0.5872 |
|  | **Debris** | Coefficient | 0.53284 | 0.28329 | 0.12245 | 1 |
|  |  | P value | 0.0107 | 0.2014 | 0.5872 |  |
| **C2D1** | **CTC** | Coefficient | 1 | 0.30776 | -0.02681 | 0.05616 |
|  |  | P value |  | 0.1747 | 0.9082 | 0.809 |
|  | **Apoptotic CTC** | Coefficient | 0.30776 | 1 | 0.26264 | 0.14769 |
|  |  | P value | 0.1747 |  | 0.2501 | 0.5229 |
|  | **CTC debris** | Coefficient | -0.02681 | 0.26264 | 1 | -0.2775 |
|  |  | P value | 0.9082 | 0.2501 |  | 0.2233 |
|  | **Debris** | Coefficient | 0.05616 | 0.14769 | -0.2775 | 1 |
|  |  | P value | 0.809 | 0.5229 | 0.2233 |  |
